# Exploring the Link Between Sound Quality Perception, Music Perception, Music Engagement, and Quality of Life in Cochlear Implant Recipients

**DOI:** 10.3390/audiolres15040094

**Published:** 2025-08-02

**Authors:** Ayşenur Karaman Demirel, Ahmet Alperen Akbulut, Ayşe Ayça Çiprut, Nilüfer Bal

**Affiliations:** 1 Vocational School of Health Services, Istanbul Okan University, Istanbul 34959, Türkiye; 2 Audiology and Speech Disorders PhD Program, Institute of Health Sciences, Marmara University, Istanbul 34854, Türkiye; a.alperenakbulut1@gmail.com; 3 Department of Audiology, Hamidiye Faculty of Health Sciences, University of Health Sciences, Istanbul 34668, Türkiye; 4 Department of Audiology, Faculty of Medicine, Marmara University, Istanbul 34899, Türkiye; aycaciprut1@yahoo.com (A.A.Ç.); fzt.niluferondag@gmail.com (N.B.)

**Keywords:** TR-MUSHRA, MuRQoL, sound quality perception, music perception, music engagement

## Abstract

**Background/Objectives**: This study investigated the association between cochlear implant (CI) users’ assessed perception of musical sound quality and their subjective music perception and music-related quality of life (QoL). The aim was to provide a comprehensive evaluation by integrating a relatively objective Turkish Multiple Stimulus with Hidden Reference and Anchor (TR-MUSHRA) test and a subjective music questionnaire. **Methods**: Thirty CI users and thirty normal-hearing (NH) adults were assessed. Perception of sound quality was measured using the TR-MUSHRA test. Subjective assessments were conducted with the Music-Related Quality of Life Questionnaire (MuRQoL). **Results**: TR-MUSHRA results showed that while NH participants rated all filtered stimuli as perceptually different from the original, CI users provided similar ratings for stimuli with adjacent high-pass filter settings, indicating less differentiation in perceived sound quality. On the MuRQoL, groups differed on the Frequency subscale but not the Importance subscale. Critically, no significant correlation was found between the TR-MUSHRA scores and the MuRQoL subscale scores in either group. **Conclusions**: The findings demonstrate that TR-MUSHRA is an effective tool for assessing perceived sound quality relatively objectively, but there is no relationship between perceiving sound quality differences and measures of self-reported musical engagement and its importance. Subjective music experience may represent different domains beyond the perception of sound quality. Therefore, successful auditory rehabilitation requires personalized strategies that consider the multifaceted nature of music perception beyond simple perceptual judgments.

## 1. Introduction

A cochlear implant (CI) is an electronic device that utilizes the tonotopic organization of the cochlea to stimulate the auditory nerve through electrodes surgically implanted within the cochlea. CI technology currently exhibits limitations in the transmission of complex stimuli, such as music. Music is formed by the organization of fundamental sound elements—including frequency, duration, intensity, and timbre—over time [1]. Various technological, biological, and acoustic constraints impact the perception of music for CI users. From a technological perspective, limitations in spectral resolution, temporal processing, and dynamic range representation pose challenges. Biologically, damage to the auditory nerve and atypical activation of the auditory cortex can further hinder music perception. Additionally, the complex nature of music, which requires the perceptual integration of rhythmic, melodic, harmonic, and timbral elements, presents acoustic challenges. These factors collectively contribute to the difficulties that CI recipients experience when processing music [2]. Due to these limitations, the ability of CI users to access and decode complex musical stimuli is negatively impacted compared to individuals with normal hearing (NH), resulting in a different perception of music compared to acoustic hearing.

CI users characterize music as discordant, lacking emotional depth, and deficient in low frequencies. They also face challenges with the genre of music, lyrics, timbre, and other aspects [3]. A significant aspect concerning the perceptual constraints of music for CI users is the perception of sound quality. Sound quality refers to how rich an auditory stimulus is perceived to be. The impairment of several auditory components in CI users may result in decreased sound quality compared to individuals with NH, negatively affecting the music listening experience [4]. Individuals with CIs generally assess the quality of musical sounds as less enjoyable compared to those with NH [5]. Almost one-third of implant users expressed a tendency to avoid music due to its unpleasant sound quality [6].

Sound quality ratings can be assessed using both objective and subjective measures. Although sound quality in CI users is assessed using visual analogue scales [7,8], these subjective assessments may be influenced by various aspects related not just to sound quality but also to the stimuli and the person giving the assessment [4]. To address the need for a novel approach that offers a more quantitative evaluation of sound quality perception in CI users, a new instrument for sound quality assessment has been developed. Cochlear Implant-Multiple Stimulus with Hidden Reference and Anchor (CI-MUSHRA) allows the assessment (via numerical ratings) of the original stimulus (“reference”) alongside its filtered variants and a significantly altered version (termed “anchor”), demonstrating the capacity to differentiate between variations in sound quality among musical stimulus versions [9].

Listening to music can provide enjoyment to the listener and may improve their quality of life [10]. The inherent constraints of CI, compounded by challenges associated with hearing loss, can result in reduced engagement in or complete avoidance of musical activities [11]. This avoidance of music, in turn, has been shown to have negative effects on quality of life [12]. Changes in listening habits and decreased enjoyment of music after cochlear implantation have also been reported in the literature [6,13]. In a study of adult CI users, 38% of participants expressed a distaste for music, while 86% had decreased listening habit ratings post-implantation [14]. The constraints of CIs regarding music extend beyond sound degradation; they may also diminish the personal and social benefits that music provides [3]. The physiological, psychological, and socio-emotional advantages of music are well established [15]. Considering that music is a significant element in human life that reflects emotions and thoughts, improving sound quality after implantation may contribute to an enhanced quality of life [16].

Music therapy can not only lead to an improvement in quality of life, but it can also support auditory rehabilitation and help CI recipients return to their social lives with higher self-esteem and greater self-confidence [17]. Previous research has examined the correlation between music and quality of life by individually assessing music scores and quality of life scores, followed by an analysis of their interrelationship [18,19]. The Music-Related Quality of Life (MuRQoL) questionnaire aims to overcome this limitation by directly evaluating the effect of music on quality of life. The MuRQoL questionnaire also includes subscales to assess music perception and music engagement [12]. In a comprehensive analysis of music assessment techniques following cochlear implantation, the authors suggested a dual methodology for evaluating the post-cochlear implant music experience, employing subjective measures via questionnaires like the MuRQoL and objective assessments through music perception tests [20].

The challenge of enhancing music perception and appreciation in CI recipients, and its consequent impact on quality of life, is well established. Accordingly, research has largely focused on identifying perceptual limitations to guide technological developments and structure effective rehabilitation programs. This approach, however, rests on the critical yet unverified assumption that enhancements in the technical perception of sound quality necessarily translate to improvements in real-world music experiences or music-related quality of life. The present study aims to address this gap by examining the relationship between CI users’ performance on a relatively objective sound quality evaluation (TR-MUSHRA) and their self-reported levels of music perception, musical engagement, and music-related quality of life (MuRQoL). By integrating these potentially distinct domains of auditory experience—objective performance-based assessment and subjective self-report—this study seeks to offer a more comprehensive and multidimensional perspective on the music experiences of CI users.

## 2. Materials and Methods

### 2.1. Participants

The control group of this study consisted of 30 adults with NH (aged 25–61 years, mean age = 34.3, SD = 9.47). A total of 30 CI users (aged 18–67 years; mean age = 37.5, SD = 13.2) who regularly attended follow-up appointments for CI programming at Marmara University Hospital were included in the CI group. The group consisted of 19 males and 11 females, including 9 individuals with prelingual hearing loss onset and 21 with postlingual hearing loss onset. Additionally, 8 participants were bimodal users, while 22 were unilateral users. All participants used Cochlear or MED-EL manufactured CIs, and the frequency maps of all users included allocations below 200 Hz. All participants had CI thresholds of 30 dB HL or better between 500 and 4000 Hz and had been using their CIs for at least 3 years. Bimodal users participated in the experiment with a hearing aid on the contralateral ear. Unilateral users had no residual hearing in the contralateral ear. Individuals with hearing loss occurring before the age of 2 were classified as prelingual users, whereas those with hearing loss occurring at or after the age of 2 were classified as postlingual users.

For NH participants, inclusion criteria included being at least 18 years of age and having hearing thresholds of 25 dB HL or better at four frequencies: 500 Hz, 1000 Hz, 2000 Hz, and 4000 Hz. None of the participants in either group had received formal music education beyond mandatory school music classes.

Before testing, the purpose and procedures of this study were thoroughly explained to all participants. This study was conducted at the Audiology Department of Marmara University and was approved by the Ethics Committee (Protocol No. 09.2022.994). Written informed consent was obtained from all participants in accordance with ethical guidelines.

### 2.2. Procedures

A data collection form, particularly designed to obtain information on hearing loss in CI users, along with their music listening behaviors, music education, enjoyment of music, demographics, and descriptive details regarding both CI users and individuals with NH, was handed out to all participants.

The Turkish version of Cochlear Implant-Multiple Stimulus with Hidden Reference and Anchor (TR-MUSHRA) test was used to evaluate differences in perceived sound quality between the reference stimulus and all tested stimuli. In addition, the Music-Related Quality of Life Questionnaire was administered to assess participants’ subjective music perception abilities, and their attitudes toward music and musical activities. To evaluate the impact of music on an individual’s quality of life, a matrix was used to cross-reference scores from the two main scales of the MuRQoL. These were the Frequency Scale (FS), which measures how often individuals perceive they can successfully perform music-related tasks, and the Importance Scale (IS), which measures how personally important those same tasks are to them. By analyzing the relationship—particularly the discrepancies—between these two scales, the direction (positive or negative) of music’s impact on quality of life was determined.

### 2.3. TR-MUSHRA (Turkish Version of Multiple Stimuli with Hidden Reference and Anchor)

MUSHRA is an open-source audio engineering software developed in 2005 for evaluating the quality differences among multiple audio sources. It has been widely adopted in the audio industry [21,22]. CI-MUSHRA, an adaptation of MUSHRA, was specifically developed for CI users [9]. It has been described as a reliable and an adaptable tool for assessing sound quality perception in both NH individuals and CI users [4].

TR-MUSHRA is the Turkish-language adaptation of CI-MUSHRA, designed to evaluate sound quality perception in native Turkish-speaking CI users. While maintaining the core features of the original MUSHRA software, the interface buttons and instructions were translated into Turkish with clear and user-friendly terminology. Furthermore, the musical stimuli in TR-MUSHRA consist of Turkish songs that are culturally relevant to the participants. This tool provides a reliable framework for assessing perceived sound quality [23].

The stimuli used in TR-MUSHRA consisted of 5 s excerpts from 20 Turkish songs. These songs, categorized into five genres (Turkish Pop, Turkish Rock, Turkish Arabesque Fantasy, Turkish Art Music, and Turkish Folk Music), were curated with the assistance of the Music Department at Marmara University’s Faculty of Fine Arts. Each song was presented in seven variations during the TR-MUSHRA test. These included five high-pass (HP) filtered versions with cutoff frequencies at 200 Hz, 400 Hz, 600 Hz, 800 Hz, and 1000 Hz. A key factor affecting sound quality perception in CI users is the poor perception of low frequencies (bass), particularly due to limited apical cochlear stimulation. This deficit can lead to decreased musical quality and aesthetic enjoyment. The reason for using filtered musical stimuli in this study is to systematically investigate the perceptual consequences of this specific limitation. Progressively removing bass frequency content via high-pass filters offers a controlled method to assess the ability of CI users to differentiate these changes [9]. Therefore, this task provides a behavioral measure of how deficits in low-frequency perception impact music quality, complementing the subjective quality of life data obtained from questionnaires. In addition, a highly distorted band-pass (BP) filtered version (1000–1200 Hz) and one unaltered reference version, identical to the original, were also presented. Rather than rating the absolute sound quality of each stimulus, participants were instructed to assess the perceived differences in sound quality between the unaltered reference stimulus and the filtered or distorted versions (200 Hz HP, 400 Hz HP, 600 Hz HP, 800 Hz HP, 1000 Hz HP, and 1000–1200 Hz BP).

Participants first listened to the reference stimulus. They then evaluated seven different versions of the stimulus, comparing each one with the reference version. Participants rated the perceived sound quality differences on a numerical scale ranging from 0 to 100, where 0 indicated the stimulus least similar to the reference and 100 indicated the stimulus most similar to the reference. They were allowed to replay the stimuli as needed. A training phase preceded the main assessment to ensure that participants understood the procedure. Once the technique was clearly comprehended, the evaluation phase started. Acoustic stimuli were delivered through a JBL Control One loudspeaker (JBL, Harman International, Stamford, CT, USA) positioned 1 m from the participant at a 0-degree azimuth within a soundproof booth. The sound pressure level (SPL) was calibrated to 65 dB using a sound level meter.

**Figure 1:**
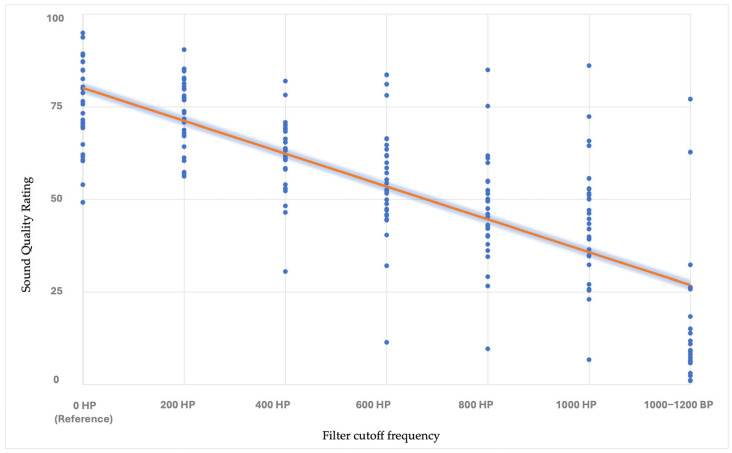
This figure shows linear regression analysis for mean sound quality ratings as a function of high-pass filter cutoff frequencies in CI users. Circles represent individual scores, while the solid line indicates the overall trend of decreasing ratings with increasing cutoff frequencies.

Each participant rated 140 instances (7 different versions of 20 musical stimuli) during the TR-MUSHRA test. To determine a single score for each participant to use in the analyses, the correlation coefficient of each sample was used as the TR-MUSHRA score. The correlation coefficient measures the relationship between the independent variable (cutoff frequency) and the dependent variable (TR-MUSHRA scores), indicating whether the relationship is positive or negative. The magnitude of the correlation coefficient reflects the strength of the relationship, with values closer to −1 signifying a stronger negative correlation. This analytical approach provides a robust metric for evaluating scores assigned to cutoff frequencies on an individual basis. It also facilitates comparisons of individual sensitivity levels among participants (see Figure 1).

### 2.4. The Music‐Related Quality of Life Questionnaire (MuRQoL)

The MuRQoL was developed as a self-administered tool for evaluating music perception skills, music experiences, and music-related quality of life among adults with CIs [12]. Its primary purpose is to provide guidance for music rehabilitation interventions. The MuRQoL was adapted into Turkish, and a validity and reliability study was conducted [24].

The MuRQoL consists of two main components: the Frequency Scale (FS) and the Importance Scale (IS). The FS evaluates music perception skills and musical experiences, while the IS assesses the importance of these skills and experiences. Both the FS and IS subscales include 18 items rated on a 5-point Likert scale (1 = never, 5 = always). The FS section of the questionnaire is divided into two subscales. The “Music Perception” (FS_MP_, questions 1–11) subscale contains 11 items evaluating music listening skills. The “Music Engagement” (FS_ME_, questions 12–18) subscale contains 7 items evaluating attitudes towards music and musical activities. The IS section of the questionnaire is also divided into two subscales. The “Music Perception” (IS_MP_, questions 1–11) subscale assesses the importance of listening skills across 11 items. The “Music Engagement” (IS_ME_, questions 12–18) subscale assesses the importance of attitudes towards music and musical activities across 7 items. Further details of the MuRQoL questionnaire can be seen in the Supplementary Materials.

The scores from each part were individually summed for the FS and the IS, then divided by the number of questions in each section or subsection (e.g., 18 for the FS total and 11 for the FS_ME_) to derive the final scores utilized for the statistical analysis. A matrix combining the FS and IS scores was used to evaluate the impact of music on quality of life [12]. For instance, when music is deemed important (high IS score) but the corresponding perception or music activity item has a low score (low FS score), a strong negative impact (SNI) of music on quality of life is expected. The SNI area indicates “critical” scores for clinical rehabilitation purposes.

### 2.5. Statistical Analysis

The data obtained in this study were statistically analyzed using IBM SPSS Statistics Version 25.0, and the results were interpreted in the context of the existing literature. Descriptive statistics were calculated to examine the distribution of the data. Normality tests were conducted to assess the data distribution, and based on the results, appropriate statistical tests were selected. Parametric tests or non-parametric tests were applied as necessary. Additionally, correlation analyses were performed depending on the data characteristics. Statistical significance was defined as *p* < 0.05 for a two-tailed test.

## 3. Results

The TR-MUSHRA test was administered to evaluate the participants’ perception of sound quality across acoustically manipulated versions of a musical stimulus. The MuRQoL questionnaire was used to assess music listening skills, attitudes toward music and music-related activities, as well as the importance attributed to these skills and activities.

There were significant differences between the NH participants and the CI users in the TR-MUSHRA scores for the reference stimulus (U = 110, *p* < 0.001), 200 Hz HP (U = 309, *p* < 0.05), 600 Hz HP (U = 276, *p* < 0.05), 800 Hz HP (U = 240, *p* < 0.05), 1000 Hz HP (U = 17,258, *p* < 0.001), and 1000–1200 Hz BP (U = 233, *p* < 0.001) stimuli, but there was no significant difference between the groups for the 400 Hz HP stimulus (U = 360, *p* > 0.05). A Kruskal–Wallis test was conducted to investigate differences in the TR-MUSHRA scores among the seven stimulus conditions. A significant effect of the filters applied to the stimuli on the sound quality scores was observed in both NH (χ^2^(6) = 194, *p* < 0.001, η^2^ = 0.929) and CI users (χ^2^(6) = 130, *p* < 0.001, η^2^ = 0.622). To further examine these differences, a post hoc Games–Howell test was performed to identify pairwise differences between stimulus conditions. NH participants were able to recognize differences in sound quality between the original stimulus and all sound versions (*p* < 0.001). In the CI group, no significant differences were found between specific pairs (reference vs. 200 Hz HP, *p* = 0.985; 400 Hz HP vs. 600 Hz HP, *p* = 0.278; 600 Hz HP vs. 800 Hz HP, *p* = 0.509; 600 Hz HP vs. 1000 Hz HP, *p* = 0.185; 800 Hz HP vs. 1000 Hz HP, *p* = 0.991). All other contrasts were significantly different (e.g., reference vs. 400 Hz HP, *p* < 0.001; reference vs. 1000–1200 Hz BP, *p* < 0.001; 600 Hz HP vs. 1000–1200 Hz BP, *p* < 0.001). These results indicate that stimulus type significantly affects the TR-MUSHRA scores. These findings suggest that CI users experienced difficulty in perceptually distinguishing between certain stimuli.

There was no significant difference between CI users and NH participants in the Importance Scale (IS_MP_ (U = 414, *p* = 0.599) and IS_ME_ (U = 346, *p* = 0.125)). However, significant differences were found in the Frequency Scale (FS_ME_ (U = 94, *p* < 0.001) and FS_MP_ (U = 274, *p* < 0.05)).

The Mann–Whitney U test revealed a statistically significant difference in the TR-MUSHRA scores between the unilateral and bimodal CI user groups (U = 33.0, *p* = 0.011, Rank Biserial Correlation = −0.625). These results indicate that the scores of individuals with bimodal CIs are significantly better than those of unilateral CI users. There is no statistically significant difference in the Frequency Scale (FS_MP_ U = 74.0, *p* = 0.526; FS_ME_ U = 58.5, *p* = 0.173) and the Importance Scale (IS_MP_ U = 83.0, *p* = 0.832; IS_ME_ U = 85.0, *p* = 0.906) between the unilateral CI users and bimodal CI users (*p* > 0.05).

There is no statistically significant difference in the TR-MUSHRA scores (U = 65.0, *p* = 0.189), the Frequency Scale (FS_MP_ U = 77.5, *p* = 0.454; FS_ME_ U = 58.0, *p* = 0.102) and the Importance Scale (IS_MP_ U = 64.0, *p* = 0.173; IS_ME_ U = 76.5, *p* = 0.427) between the prelingual and postlingual hearing loss groups (*p* > 0.05).

The correlation analysis did not reveal any significant correlations between the TR-MUSHRA scores and the Frequency Scale (FS_MP_ rs = −0.002, *p* = 0.991; FS_ME_ rs = −0.280, *p* = 0.134) and the Importance Scale (IS_MP_ rs = 0.068, *p* = 0.722; IS_ME_ rs = −0.198, *p* = 0.295) scores in CI users. Similarly, the correlation analysis did not reveal any significant correlations between the TR-MUSHRA scores between the Frequency Scale (FS_MP_ rs = −0.018, *p* = 0.924; FS_ME_ rs = −0.280, *p* = 0.248) and the Importance Scale (IS_MP_ rs = 0.312, *p* = 0.093; IS_ME_ rs = −0.05, *p* = 0.756) scores in NH participants. These findings suggest that the TR-MUSHRA scores do not exhibit meaningful relationships with any of the other MuRQoL questionnaire subscales in this study.

When examining the relationship between the responses to each question in the MuRQoL (a total of 36 questions across 2 subscales) and the participants’ TR-MUSHRA scores, significant correlations were found in both groups. To control for the increased risk of Type I error due to multiple comparisons across the subscales, a Bonferroni correction was applied (the adjusted significance threshold was set at *p* < 0.0014). These associations did not reach statistical significance after adjusting for multiple comparisons using the Bonferroni correction.

**Figure 2:**
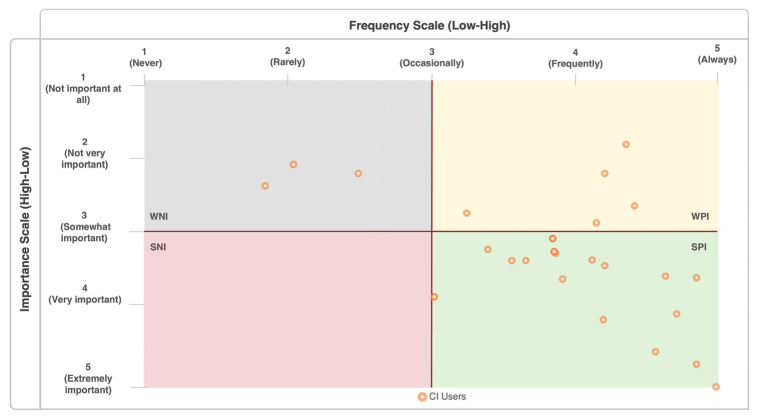
Matrix of music-related quality of life for CI users. Each circle represents an individual participant’s response. The colored quadrants illustrate the type of impact: Strong Positive Impact (SPI, green, n = 22), Weak Positive Impact (WPI, yellow, n = 5), Strong Negative Impact (SNI, red, n = 0), and Weak Negative Impact (WNI, grey, n = 3).

Among the CI user group, none of the participants showed a strong negative impact (SNI); participants showing a strong positive impact (SPI) constituted the majority with 22 individuals. The number of participants showing a weak negative impact (WNI) was 3, while the number of participants showing a weak positive impact (WPI) was 5. In the NH group, all participants were in the SPI area (see Figure 2).

The Mann–Whitney U test was conducted to examine whether there was a significant difference in the TR-MUSHRA scores between the group with a SPI of music on quality of life and the other groups. There was no statistically significant difference between the SPI group and the other group (U = 66.5, *p* = 0.324).

## 4. Discussion

### 4.1. Comparison of NH Individuals and CI Users

Significant differences between the NH and CI groups for stimulus scores on TR-MUSHRA (reference, 200 Hz HP, 600 Hz HP, 800 Hz HP, 1000 Hz HP, and 1000–1200 Hz BP) suggest that CI users have a reduced ability to perceive sound quality changes produced by these filters compared to NH individuals. The observation that NH participants consistently assigned higher sound quality ratings to the reference stimulus compared to all acoustically manipulated versions, whereas CI users did not exhibit this pattern, suggests a reduced sensitivity to perceived sound quality differences among CI users. Specifically, CI users provided comparable ratings for certain filtered stimuli, reflecting a limited ability to differentiate between subtle variations in perceived sound quality.

Some factors that limit the spectral resolution capabilities of CIs are frequently cited in the literature. One of these is that CI electrodes have a limited number of electrodes and are inherently limited by the design of the electrode array. Postlingual deafened CI users must adapt to both spectrally reduced and shifted auditory inputs, which can hinder their ability to perceive fine spectral changes [25]. Furthermore, while there may be some improvement in spectral ripple discrimination ability over time, this improvement is often inconsistent and does not always translate to enhanced speech recognition or music perception [26]. This may be attributed to the fact that the CI users in our study, irrespective of their implant experience or training, exhibited reduced sensitivity to variations in perceived sound quality, likely due to the inherent limitations in spectral resolution.

Similar to the findings of our previous study, the fact that there was no difference in the MuRQoL importance subscale scores between the NH and CI groups indicates that music has a similar level of importance, regardless of hearing related disadvantages [27]. However, although music has a similar importance for both groups, music perception and music activities measured by MuRQoL differ significantly between the groups. This finding shows the effect of hearing on practical experiences related to music even if the importance level of music does not change.

### 4.2. Within-Group Comparisons for CI Users

Compared to unilateral CI use, bimodal CI use has the advantages of providing a richer and more complete auditory signal, increasing speech perception in both quiet and noisy environments, and improving sound localization [28,29,30,31].

In this study, the significant difference in the TR-MUSHRA scores between bimodal and unilateral users may be due to the fact that the acoustic input provided a richer and more complete auditory signal in bimodal users, enhancing their perception of differences in sound quality. The integration of acoustic and electric signals in bimodal hearing has been shown to mitigate the limitations associated with unilateral CI use. Bimodal users often experience improved sound quality and clarity, which can be attributed to the complementary nature of the two types of auditory input [32].

However, the lack of significant differences in MuRQoL frequency and importance subscales indicates that although bimodal CI users may have a perceptual advantage in terms of sound quality discrimination, the importance they attach to perception of music, participation in musical activities and music are similar to those of unilateral CI users. The reason for this was thought to be that factors beyond the perception of sound quality may also be effective in shaping the perception of music, participation in music activities, and the importance given to music-related experiences.

When it is assumed that postlingual CI users have previous NH experience and this may provide an advantage in the perception of musical sound quality, the lack of difference between groups in the TR-MUSHRA and MuRQoL scales may be unexpected. However, for individuals with postlingual hearing loss, various factors influence skills such as speech and music perception and sound quality perception following cochlear implantation. These factors include the onset timing of hearing loss, its etiology and progression, hearing aid utilization post-hearing loss and the benefits derived from it, the timing of cochlear implantation, the duration and severity of auditory deprivation, implantation success, and auditory training conducted before and after implantation. Moreover, cross-modal plasticity observed in CI users may also contribute to the difficulties in processing fine spectral information. The reorganization of the auditory cortex in response to visual stimuli can hinder the effective recruitment of auditory processing pathways, thereby impacting the perception of auditory details [33]. This maladaptive plasticity may be particularly pronounced in individuals who were prelingually deafened, as their auditory cortices may have undergone significant reorganization prior to implantation [34]. Overall, the absence of a significant difference between CI users with prelingual and postlingual hearing loss in both evaluations indicates that this outcome cannot be attributed solely to the timing of hearing loss onset; rather, other factors may play a more substantial role in influencing subjective musical perception, sound quality perception, and importance among CI users.

While it is acknowledged that factors such as age, duration of CI use, CI modality, the unequal distribution of subgroups, and the small sample size may influence these results, conducting an analysis that simultaneously accounts for all these variables while focusing solely on the timing of hearing loss presents significant challenges. The multifactorial nature of CI outcomes, combined with the inherent variability among individuals, makes it difficult to isolate the effect of a single factor such as the onset of hearing loss, without introducing confounding variables or requiring an exceptionally large and balanced sample size.

### 4.3. Relationship Between TR-MUSHRA and MuRQoL

The lack of correlation between the TR-MUSHRA scores and the MuRQoL scores in NH participants and CI users suggests that the perceived sound quality, as measured by the TR-MUSHRA test, may not be directly related to individuals’ subjective musical perception, frequency of music engagement, or the importance they place on music. The similar lack of correlations observed in both CI users and NH participants suggests that the absence of association between sound quality discrimination and subjective musical perception, musical interaction, and importance is a general condition rather than one specific to CI users. Therefore, rather than assuming that there is no relationship between these two assessment domains and that they are independent or distinct domains, it was thought that the lack of a significant relationship may be related to the fact that the sound quality measure provided by TR-MUSHRA is an output that may not be captured by subjective assessment scales [35]. It was observed in our study that most of the NH participants tended to give high scores in the MuRQoL questionnaire, while some participants performed below average in the TR-MUSHRA test. Regardless of TR-MUSHRA performance, with regard to self-reporting having the potential to either overestimate or underestimate the actual degree of perceptual accuracy, the tendency of almost all NH participants to give high scores in our study was thought to support the fact that there was no correlation between the two assessment tools in the NH group and that TR-MUSHRA provides a more objective and higher assessment than the questionnaire. Conversely, an individual who is sensitive to variations in perceived sound quality may rarely engage with music or assign little importance to it.

To fully understand musical engagement, it is essential to adopt a holistic perspective that considers a range of factors. Bronfenbrenner’s Bioecological Model of Human Development offers a valuable framework for this. According to this model, an individual’s engagement with music is not determined by a single element but is shaped by a series of multi-layered and interactive contexts. These contexts range from individual experiences to environmental influences and socio-political factors [36,37].

Framed within Bronfenbrenner’s model, the TR-MUSHRA test assesses a single, specific component of the individual’s microsystem. On the other hand, subjective questionnaires like the MuRQoL can, by their nature, reflect a more holistic experience. In light of this finding, the lack of correlation can be considered to stem not from an inadequacy of the measurement tools, but rather from their targeting of different layers of human experience: one measures a fundamental perceptual skill at a micro-level, while the other potentially captures the personal meaning of that experience at a holistic level.

### 4.4. MuRQoL Matrix

CI users often experience significant challenges in music perception, which can be attributed to several factors related to the limitations of current CI technology and the inherent complexities of musical sound [2]. Despite these challenges, some CI users report enjoyment of music and the ability to recognize certain melodies, albeit at a reduced level compared to their NH peers [13,16]. The music experiences of CI users are influenced not only by factors such as their auditory profiles and the characteristics of the auditory signal, but also by the dynamic interplay of their expectations, self-management and coping strategies, personal effort and attitudes, as well as their social environment and broader contextual surroundings [11]. A successful relationship with music also hinges on the user’s ability to manage expectations and employ active coping strategies. This may involve cultivating a strong sense of self-efficacy—a belief in one’s capability to overcome challenges—and using specific listening tactics, such as focusing on lyrics, utilizing vibrotactile cues, or experimenting with different musical genres to find what is most accessible. The user’s attitude may also be critical; those who maintain a positive affective attitude (a genuine love for music) and an instrumental attitude (a belief that music is beneficial for well-being) may be more likely to persevere. This internal resilience can be significantly bolstered or hindered by the external social environment. For children, the importance parents place on music can be a direct predictor of their involvement, while for adolescents, peer acceptance can be a powerful motivator [38]. This entire ecosystem of personal mindset and social support may play a role in the context in which a CI user either successfully navigates the challenges of music perception or withdraws from it.

Similar to the findings obtained in our previous study on music-related quality of life in CI users, in this study, the fact that most of the CI users were in the SPI area in the matrix indicates that CI users can still establish a positive relationship with music despite perceptual limitations [27]. It is thought that the finding related to the quality of life matrix shows that music can still contribute positively to the quality of life of CI users. In addition, despite the disadvantages and limitations related to the device and hearing loss, the fact that none of the CI users were in the SNI area may indicate that CI users may have adjusted their expectations and importance levels about music according to their current functional status. Listening to music often requires effort and active engagement, and the level of satisfaction is influenced by the listener’s expectations and their ability to manage or adapt to the listening situation. These factors may enable them to achieve satisfactory outcomes despite challenging listening conditions and may even lead them to report their experiences as enjoyable [11]. For many CI users, listening to music is not a passive activity but an effortful process of “learning to listen” that requires conscious intentionality and persistence. The satisfaction derived is therefore not solely dependent on the fidelity of the auditory signal; it is deeply intertwined with the listener’s expectations and adaptive strategies [39,40]. Successful listeners may learn to shift their attention away from degraded elements like pitch and instead focus on well-preserved cues such as rhythm and tempo. This cognitive adaptation may allow them to perceive higher level musical structures, such as patterns of tension and release, in a manner remarkably similar to NH listeners, even when the underlying melodic information is distorted. This ability to find meaning within the available signal, combined with the intrinsic rewards of music—such as emotional aspects, relaxation, and a connection to personal identity—may enable users to achieve satisfactory and genuinely enjoyable experiences despite significant perceptual deficits [5,41,42].

Apart from this, it may be due to the fact that CI users have a level of music perception that prevents them from completely distancing themselves from music. We believe that these findings may also be related to the technological developments in recent years and the increasing awareness of music rehabilitation. This growing awareness may encourage patients to seek solutions rather than completely distancing themselves from music. For instance, a recent non-randomized clinical trial demonstrated that an accessible, home-based music rehabilitation program using a mobile phone application can lead to subjective improvements in music perception and overall quality of life for CI users. In total, 57% of participants in the experimental group reported subjective improvements after the training program [43].

A noteworthy finding of our study is that none of the CI participants exhibited a strong negative impact (SNI) in the matrix assessing music-related quality of life. While our findings demonstrate that music-related quality of life is generally positive among CI users, it is important to acknowledge that individual experiences may be influenced by broader psychosocial factors. Despite achieving significant gains in hearing abilities, CI and hearing aid users may still face specific challenges related to body image, social interaction, and overall psychological well-being. The visibility of hearing devices and the associated stigma are critical factors influencing users’ self-perception and social integration. Prior research has reported a significant association between body image perception and quality of life, highlighting that stigma related to hearing loss and hearing device use can negatively affect quality of life, and that fostering a positive body image is vital for enhancing individuals’ overall well-being [44,45,46,47,48,49]. Although our study did not collect direct data on psychosocial factors, the findings suggest that music may serve as a domain that is more resilient to social stressors. This potential interaction between personal, positive musical experiences and challenging social contexts may serve as a valuable starting point for future research exploring how musical engagement contributes to broader psychosocial resilience in CI users.

The small number of participants in the WNI and WPI domains (3 and 5 people, respectively) shows the heterogeneous nature of the group. This heterogeneity highlights the importance and need of individualized music rehabilitation programs for CI users [27].

The music-related quality of life in CI users may be influenced by a multitude of factors, including musical background, rehabilitation practices, psychological state, and the inherent limitations of CI technology. The fact that no significant difference was observed when comparing the TR-MUSHRA scores of CI users in the SPI domain with the TR-MUSHRA scores of CI users in other domains suggests that the ability to discriminate sound quality may not be the main or decisive factor among the factors determining music-related quality of life. However, it is important to acknowledge that the SPI group comprised 22 people, while the WNI and WPI groups consisted of a combined total of 8 individuals (3 and 5, respectively), which may compromise the reliability and validity of the comparison. The fact that all participants in the NH group were in the SPI domain is an expected finding from our previous study and confirms the strong positive effect of music on quality of life in the NH population [27].

The findings of this study inevitably have limitations, which may affect the generalizability of the results to broader applications and future research. The key limitations of our study are as follows:

(a) A significant limitation of this study is the small sample size and the considerable heterogeneity within the CI group. This group included pre and postlingual users, as well as unilateral and bimodal listeners. These distinct subgroups may have different auditory histories and perceptual abilities, which could substantially influence their musical experiences and expectations. Our sample size limits the statistical power of subgroup analyses, particularly for the heterogeneous CI group. Given that our sample size was insufficient for reliable subgroup comparisons, our findings must be interpreted with this significant caveat in mind. Future research with larger, more homogeneous cohorts is essential to disentangle the effects of these important user-related variables.
(b) The music perception of CI users is influenced not only by the technical features of their devices but also by individual listening habits, environmental factors, and daily life experiences. This study may not have fully accounted for the individual differences that impact the musical experiences of CI users. Future research should adopt a more comprehensive approach to explore the effects of individual factors on the perception and evaluation of music in greater detail. Similarly, potential confounding variables such as prior musical training, daily listening habits, and the socio-emotional context of music engagement were not controlled for in this study and should be considered in future research to provide a more comprehensive model of music-related quality of life.
(c) The TR-MUSHRA test is limited to evaluating the perceptual dimension of sound quality and does not encompass higher-order musical constructs such as melody, harmony, or rhythm. Consequently, the conclusions drawn from this study pertain specifically to the domain of sound quality and should not be extrapolated to general music perception. Additionally, a methodological limitation of this study is the use of a fixed presentation order in the TR-MUSHRA test, as the stimuli were not randomized, potentially introducing order effects that may have biased participant ratings. Furthermore, the cross-sectional nature of the study design limits any inference of causality between objective sound quality perception and self-reported music-related quality of life.

## 5. Conclusions

In conclusion, while TR-MUSHRA serves as a relatively quantitative measure of musical sound quality discrimination, our findings indicate that it is not directly related to self-reported musical perception skills, musical experiences, or the importance attributed to these skills and experiences. This lack of correlation may stem from the inherent differences in the evaluation methods of TR-MUSHRA and the MuRQoL scale. TR-MUSHRA provides a relatively objective, standardized assessment of sound quality discrimination, whereas the MuRQoL scale reflects subjective perceptions influenced by individual preferences, experiences, and contextual factors.

The sound quality perception measured by TR-MUSHRA may not directly influence musical perception skills, experiences, or their perceived importance. This highlights the need to consider a more holistic framework. Moreover, the findings emphasize the importance of considering the multifactorial nature of music perception and the subjective experience of music, particularly in CI users. Despite the perceptual limitations associated with CIs, CI users continue to engage with music and derive meaning and satisfaction from it. This suggests that music perception and its importance to quality of life extend beyond measurable auditory skills, highlighting the need for individualized rehabilitation strategies that address not only the perceptual challenges but also the broader subjective and contextual aspects of music for the listener. Rehabilitation programs may benefit from adopting individualized and multidimensional approaches that go beyond perceptual training and incorporate the listener’s emotional, social, and contextual relationship with music. Interventions that address expectation management, motivation, and personal meaning—possibly through counseling or personalized music activities—may enhance engagement and outcomes. For example, future protocols could incorporate the listener’s emotional and social context by including psychosocial support to help users manage expectations and build self-efficacy, which are critical for sustained engagement. Given the reported “spillover” effect of music training on improving speech-in-noise perception and music perception, these programs should be considered a core component of aural rehabilitation for all users, not just an optional extra. Similarly, these findings should inform future CI signal processing strategies to move toward greater personalization. Advancements like image-guided CI programming, which uses post-operative imaging to create an anatomy-based frequency map, represent a promising pathway to mitigate tonotopic mismatch and improve pitch perception, ultimately helping to bridge the gap between perceptual capability and subjective musical enjoyment.

## Data Availability

The data presented in this study are available on request from the corresponding author.

## Supplementary Materials

The following supporting information can be downloaded at https://www.mdpi.com/article/10.3390/audiolres15040094/s1, The MuRQoL questionnaire can be seen in the Supplementary Digital Content.

## Abbreviations

The following abbreviations are used in this manuscript:

CI	Cochlear implant
MUSHRA	Multiple Stimulus with Hidden Reference and Anchor
CI-MUSHRA	Cochlear Implant-Multiple Stimulus with Hidden Reference and Anchor
TR-MUSHRA	Turkish Version of Multiple Stimulus with Hidden Reference and Anchor
QoL	Quality of Life
MuRQoL	Music-related quality of life
NH	Normal hearing
SD	Standard deviation
FS	Frequency Scale
IS	Importance Scale
MP	Music perception
ME	Music engagement
WNI	weak negative impact
WPI	weak positive impact
SNI	strong negative impact
SPI	strong positive impact
