# Supplementary material for: Exploring the Link Between Sound Quality Perception, Music Perception, Music Engagement, and Quality of Life in Cochlear Implant Recipients
Source: Audiol Res. 2025 Aug 2;15(4):94. doi: 10.3390/audiolres15040094 (PMC12382851; doi:10.3390/audiolres15040094)
Supplement: Supplementary file 1 [file audiolres-15-00094-s001.zip › audiolres-3733243-supplementary.pdf]

The original English version of the Music-Related Quality of Life (MuRQoL) Questionnaire is presented in the following.

If you are willing to use the MuRQoL questionnaire (English version) in your study, please get in touch with Giorgos Dritsakis (gd.dritsakis@gmail.com).

Please cite the MuRQoL questionnaire (English version) as:

Dritsakis, G., Van Besouw, R. M., Kitterick, P., & Verschuur, C. A. (2017). A music-related quality of life measure to guide music rehabilitation for adult cochlear implant users. American journal of audiology, 26(3), 268-282.

<u>The ‘Music-Related Quality of Life’ measure (MuRQoL-En)</u>

Part I

This part of the questionnaire asks you about your music listening abilities, attitudes towards music and musical activities. Please answer the following questions by using one of the options: **<u>1: Never, 2: Rarely, 3: Occasionally, 4: Frequently, 5: Always, N/A: Not Applicable</u>**.

MUSIC PERCEPTION	1	2	3	4	5	N/A
1. Can you distinguish different rhythms in music?						
2. Can you follow the melody in music (i.e. follow the melody of a song or a familiar tune)?						
3. Can you hear differences in musical tone (i.e. how high or low music is)?						
4. Can you recognize the words in songs?						
5. Can you recognize the sounds of different musical instruments?						
6. Can you hear the meaning of music (i.e. the emotion, why it was created or what message it is trying to get across)?						
7. Can you hear music without effort or having to concentrate?						
8. Can you recognize familiar music (e.g. a song, singer or tune)?						
9. Can you judge the quality of a musical performance (e.g. singing or musical instrument playing)?						
10. Do you feel confident that you hear music like other people do?						
11. Does music sound in tune?						

MUSIC ENGAGEMENT	1	2	3	4	5	N/A
12. Do you enjoy music in noisy environments when no visual cues are available (e.g. at a party, at a restaurant or in the car over the engine/ road noise)?						
13. Do you enjoy music on TV, laptop, tablet or on the phone?						
14. Do you choose to have music on in the background while doing something else (e.g. while reading, painting, doing gardening, exercising or just relaxing)?						
15. Do you listen to music while travelling (e.g. in the car)?						
16. Do you choose to listen to new music (i.e. music that you have not heard before)?						
17. Do you attend public music events (e.g. musicals, concerts or music festivals)?						
18. Do you sing, play a musical instrument or whistle?						

© 2025 Giorgos Dritsakis

Part II

This part of the questionnaire asks you how importance the above music listening abilities, attitudes towards music and musical activities are for you. Please answer the following questions by using one of the options: **1: Not important at all**, **2: Not very important**, **3: Somewhat important**, **4: Very important**, **5: Extremely important**, **N/A: Not Applicable**.

MUSIC PERCEPTION	1	2	3	4	5	N/A
1. How important is it for you to be able to distinguish different rhythms in music?						
2. How important is it for you to be able to follow the melody in music (i.e. follow the melody of a song or a familiar tune)?						
3. How important is it for you to be able to hear differences in musical tone (i.e. how high or low music is)?						
4. How important is it for you to be able to recognize the words in songs?						
5. How important is it for you to be able to recognize the sounds of different musical instruments?						
6. How important is it for you to be able to hear the meaning of music (i.e. the emotion, why it was created or what message it is trying to get across)?						
7. How important is it for you to be able to hear music without effort or without having to concentrate?						
8. How important is it for you to be able to recognize familiar music (e.g. a song, singer or tune)?						
9. How important is it for you to be able to judge the quality of a musical performance (e.g. singing or musical instrument playing)?						
10. How important is it for you to feel confident that you hear music like other people do?						
11. How important is it for you to hear music that sounds in tune?						

MUSIC ENGAGEMENT	1	2	3	4	5	N/A
12. How important is it for you to enjoy music in noisy environments when no visual cues are available (e.g. at a party, at a restaurant or in the car over the engine/road noise)?						
13. How important is it for you to enjoy music on TV, laptop, tablet or on the phone?						
14. How important is it for you to have music on in the background while doing something else (e.g. while reading, painting, doing gardening, exercising or just relaxing)?						
15. How important is it for you to listen to music while travelling (e.g. in the car)?						
16. How important is it for you to listen to new music (i.e. music that you have not heard before)?						
17. How important is it for you to attend public music events (e.g. musicals, concerts or music festivals)?						
18. How important is it for you to sing, play a musical instrument or whistle?						

© 2025 Giorgos Dritsakis

The Turkish version of the Music-Related Quality of Life Questionnaire that was used in this study is presented in the following.

If you are willing to use the MuRQoL questionnaire (Turkish version) in your study, please get in touch with Ahmet Alperen Akbulut (ahmetalperen.akbulut@sbu.edu.tr).

Please cite the MuRQoL questionnaire (Turkish version) as:

Akbulut, A.A., Çiprut, A., Akdeniz, E. et al. Translation and validation of the music-related quality of life questionnaire for adults with cochlear implant in Turkish language. Eur Arch Otorhinolaryngol 279, 685–693 (2022). https://doi.org/10.1007/s00405-021-06693-w

‘Müzikle İlgili Yaşam Kalitesi’ Anketi (MuRQoL-Tr)

Bölüm I

Anketin bu bölümünde müzik dinleme becerileriniz, müziğe ve müzik etkinliklerine yönelik tutumlarınız sorgulanmaktadır. Lütfen aşağıdaki sorulara şu seçeneklerden birini kullanarak cevap veriniz: **1: Hiç**, **2: Nadiren**, **3: Ara Sıra**, **4: Sık Sık**, **5: Her Zaman**, **U/D: Uygun Değil**.

MÜZİK ALGISI	1	2	3	4	5	U/D
1. Müzikteki farklı ritimleri ayırt edebiliyor musunuz?						
2. Müzikteki melodiyi takip edebiliyor musunuz (Ör: Bir şarkının ya da tanıdığınız bir ezginin melodisini takip etmek)?						
3. Müziğin tonundaki değişiklikleri duyabiliyor musunuz (Ör: Müziğin ne kadar alçak ya da yüksek perdeden olduğunu)?						
4. Şarkılardaki sözleri anlayabiliyor musunuz?						
5. Farklı müzik enstrümanlarının seslerini tanıyabiliyor musunuz?						
6. Müziğin anlamını duyabiliyor musunuz (Ör: Duyguyu, neden yazıldığını ya da hangi mesajı vermeye çalıştığını)?						
7. Çaba göstermeden ya da konsantre olmak zorunda kalmadan müziği duyabiliyor musunuz?						
8. Aşina olduğunuz müzikleri tanıyabiliyor musunuz (Ör: Bir şarkıyı, bir şarkıcıyı ya da bir ezgiyi)?						
9. Bir müzikal performansın kalitesini değerlendirebilir misiniz (Ör: Şarkı söylenmesi ya da bir müzik enstrümanının çalınması)?						
10. Müziği diğer insanlar gibi duyduğunuzdan emin misiniz?						
11. Müziği ahenkli duyuyor musunuz?						

MÜZİK ETKİNLİKLERİ	1	2	3	4	5	U/D
12. Görsel ipuçları olmadığında gürültülü ortamlarda müzik dinlemekten keyif alıyor musunuz (Ör: Bir partide, bir restoranda ya da motorun çalıştığı/yol gürültüsünün olduğu bir arabada)?						
13. TV, bilgisayar, tablet veya telefon ile müzik dinlemekten keyif alıyor musunuz?						
14. Başka bir şey yaparken arka planda müzik olmasını tercih ediyor musunuz (Ör: Okurken, resim yaparken, bahçeyle uğraşırken, egzersiz yaparken ya da sadece dinlenirken)?						
15. Seyahat ederken müzik dinliyor musunuz (Ör: Arabadayken)?						
16. Yeni müzikleri dinlemeyi tercih ediyor musunuz (Ör: Daha önce duymadığınız bir müziği)?						
17. Toplumsal müzik etkinliklerine katılıyor musunuz (Ör: Müzikallere, konserlere ya da müzik festivallerine)?						
18. Şarkı söylüyor musunuz, bir müzik enstrümanı ya da ıslık çalıyor musunuz?						

© 2025 Giorgos Dritsakis (Turkish Translation, Marmara University)

Bölüm II

Anketin bu bölümünde, yukarıda belirtilen müzik dinleme becerilerinin, müzik ve müzik etkinliklerine yönelik tutumların sizin için ne kadar önemli olduğu sorgulanmaktadır. Lütfen aşağıdaki sorulara şu seçeneklerden birini kullanarak cevap veriniz: **1: Hiç Önemli Değil**, **2: Çok Önemli Değil**, **3: Biraz Önemli**, **4: Çok Önemli**, **5: Fazlasıyla Önemli**, **U/D: Uygun Değil**.

MÜZİK ALGISI	1	2	3	4	5	U/D
1. Müzikteki farklı ritimleri ayırt edebilmek sizin için ne kadar önemlidir?						
2. Müzikteki melodiyi takip edebilmek sizin için ne kadar önemlidir (Ör: Bir şarkının ya da tanıdığınız bir ezginin melodisini takip etmek)?						
3. Müziğin tonundaki değişiklikleri duyabilmek sizin için ne kadar önemlidir (Ör: Müziğin ne kadar alçak ya da yüksek perdeden olduğunu)?						
4. Şarkılardaki sözleri anlayabilmek sizin için ne kadar önemlidir?						
5. Farklı müzik enstrümanlarının seslerini tanıyabilmek sizin için ne kadar önemlidir?						
6. Müziğin anlamını duyabilmek sizin için ne kadar önemlidir (Ör: Duyguyu, neden yazıldığını ya da hangi mesajı vermeye çalıştığını)?						
7. Çaba göstermeden ya da konsantre olmak zorunda kalmadan müziği duyabilmek sizin için ne kadar önemlidir?						
8. Aşina olduğunuz müzikleri tanıyabilmek sizin için ne kadar önemlidir (Ör: Bir şarkıyı, bir şarkıcıyı ya da bir ezgiyi)?						
9. Bir müzikal performansın kalitesini değerlendirebilmek sizin için ne kadar önemlidir (Ör: Şarkı söylenmesi ya da bir müzik enstrümanının çalınması)?						
10. Müziği diğer insanlar gibi duymak sizin için ne kadar önemlidir?						
11. Ahenkli bir müziği duymak sizin için ne kadar önemlidir?						

MÜZİK ETKİNLİKLERİ	1	2	3	4	5	U/D
12. Görsel ipuçları olmadığında gürültülü ortamlarda müzik dinlemekten keyif alabilmek sizin için ne kadar önemlidir (Ör: Bir partide, bir restoranda ya da motorun çalıştığı/yol gürültüsünün olduğu bir arabada)?						
13. TV, bilgisayar, tablet veya telefon ile müzik dinlemekten keyif alabilmek sizin için ne kadar önemlidir?						
14. Başka bir şey yaparken arka planda müzik olması sizin için ne kadar önemlidir (Ör: Okurken, resim yaparken, bahçeyle uğraşırken, egzersiz yaparken ya da sadece dinlenirken)?						
15. Seyahat ederken müzik dinlemek sizin için ne kadar önemlidir (Ör: Arabadayken)?						
16. Yeni müzikleri dinlemek sizin için ne kadar önemlidir (Ör: Daha önce duymadığınız bir müziği)?						
17. Toplumsal müzik etkinliklerine katılmak sizin için ne kadar önemlidir (Ör: Müzikallere, konserlere ya da müzik festivallerine)?						
18. Şarkı söylemek, bir müzik enstrümanı ya da ıslık çalmak sizin için ne kadar önemlidir?						

© 2025 Giorgos Dritsakis (Turkish Translation, Marmara University)
